# Viral infections and immune modulation in bladder cancer: implications for immunotherapy

**DOI:** 10.37349/etat.2025.1002311

**Published:** 2025-04-24

**Authors:** Lívia Bitencourt Pascoal, Mehrsa Jalalizadeh, Gabriela Barbosa, Andrea Nazare Monteiro Rangel da Silva, Maria Alice Freitas Queiroz, Ekaterina Laukhtina, Shahrokh F. Shariat, Alessandra Gambero, Leonardo O. Reis

**Affiliations:** Alma Mater Studiorum Università di Bologna, Italy; ^1^UroScience, State University of Campinas, Campinas 13083-970, Brazil; ^2^ImmunOncology, Pontifical Catholic University of Campinas, Campinas 13060-904, Brazil; ^3^INCT UroGen, National Institute of Science, Technology and Innovation in Genitourinary Cancer (INCT), Campinas 13087-571, Brazil; ^4^Laboratory of Virology, Institute of Biological Sciences, Federal University of Pará, Belém 66075-110, Brazil; ^5^Department of Urology, Comprehensive Cancer Center, Medical University of Vienna, 1090 Vienna, Austria; ^6^Hourani Center for Applied Scientific Research, Al-Ahliyya Amman University, Amman 19328, Jordan; ^7^Department of Urology, University of Texas Southwestern Medical Center, Dallas, TX 75390, USA; ^8^Department of Urology, Weill Cornell Medical College, New York, NY 10065, USA

**Keywords:** Virome, senescence, exhaustion, urothelial carcinoma, immune response

## Abstract

This review explores the intricate relationship between viral infections and Bacillus Calmette-Guerin (BCG) efficacy, emphasizing immune modulation mechanisms that may influence treatment outcomes. Since its introduction in 1976, intravesical BCG has been a cornerstone in managing non-muscle invasive bladder cancer (NMIBC) after transurethral resection of bladder tumors (TURBT). Despite its success, variability in response rates suggests that host immune status, influenced by persistent infections, immunosenescence, and antigenic overload, may play a crucial role in therapeutic effectiveness. Chronic viral infections can modulate T cell responses, leading to immune exhaustion and impaired antitumor immunity. This review discusses the interplay between viral antigenic load, immune dysfunction, and tumor microenvironment remodeling, highlighting their potential impact on immunotherapies. By integrating insights from virome analysis, immune profiling, and tumor characterization, this review proposes personalized strategies to enhance immunotherapy efficacy. A deeper understanding of viral-induced immune dysregulation may improve prognostic assessment, optimize treatment protocols, and reduce healthcare costs associated with bladder cancer. Future research should focus on targeted interventions to mitigate the immunosuppressive effects of chronic infections, ultimately improving patient outcomes in NMIBC management.

## Introduction

Cancer is a complex disease primarily driven by genetic predispositions and environmental factors. By disrupting cellular homeostasis, these elements promote the uncontrolled proliferation of abnormal cells [1]. Several risk factors contribute to the development of bladder cancer (BC), including advanced age, male sex, smoking, and chronic bladder inflammation. Exposure to aromatic amines, polycyclic hydrocarbons, heavy metals, and ionizing radiation increases the likelihood of disease onset. These factors, often in combination, play a crucial role in urothelial carcinogenesis, especially in patients with genetic predisposition [2].

BC ranks as the 6th most diagnosed cancer in males and the 10th overall [3]. Approximately 90% of BC cases are urothelial carcinomas, which develop in the cells lining the bladder. Urothelial carcinomas of the bladder are categorized into non-muscle invasive BC (NMIBC; stages 0–I), muscle-invasive BC (stages II and III), and metastatic disease (stage IV). Survival rates vary significantly depending on the stage at diagnosis, with approximately 95% survival for stage I and around 50% for stage III [4].

Since 1976, intravesical Bacillus Calmette-Guerin (BCG) therapy has been used as an immunotherapy for NMIBC following transurethral resection of bladder tumors (TURBT). BCG therapy activates the immune system in the bladder, inducing innate and adaptive immune responses. Moreover, evidence suggests that BCG directly affects the tumor microenvironment (TME) [5, 6]. A meta-analysis of individual patient data comparing intravesical maintenance chemotherapy and intravesical BCG found that BCG use was associated with a 32% reduction in the risk of recurrence [7].

In the immunogenic context of cancer, many different molecular and cellular mechanisms contribute to the failure of immunological responses that could lead to tumor eradication. These mechanisms include immune-suppressive networks that impair the function of immune cells, allowing tumors to evade detection and destruction. Environmental pressures, including chronic infections and the natural aging process, can drive such networks [8]. While genetic and ecological aspects of oncogenesis are well-documented, it is also recognized that certain microorganisms, including viruses, play a significant role in cancer development. The role of the virome, the collection of viruses within a host organism, has, therefore, emerged as a critical but still underexplored factor in cancer biology [9].

This work explores the intricate relationship between the virome and cancer, focusing on how latent viral infections, viral loads, and chronic diseases can interfere with cellular processes and the immune response. By investigating these interactions, we can gain insights into the role of the virome in cancer development and progression, potentially uncovering new therapeutic targets for improving cancer treatment and prevention.

## Viral-induced immune modulations vs. immunotherapy

In 1908, a Viennese doctor reported the disappearance of the reaction to the tuberculin skin test during active measles infection [10]. This early evidence of viral-induced immune suppression is highly relevant to our investigation, as the immune response to tuberculin is mediated by delayed-type hypersensitivity to mycobacterial components, a key mechanism in BCG immunotherapy for BC [11].

Interestingly, while this disappearance of the reaction is temporary, it persists for weeks after the resolution of measles symptoms. In 1966, Kipps et al. [12] described other infectious diseases that similarly, though less potently, diminish the immune reaction to tuberculin. These diseases include influenza, chickenpox, infectious mononucleosis, and rubella.

One could hypothesize that BCG immunotherapy might be less effective if administered during the convalescence period of a viral infection. However, the human immune system is far from simple. Numerous studies on viral-induced immune modulations have demonstrated that viruses can activate and suppress immune responses [10].

Two factors explain this ostensible paradox. First, the complexity of the human immune system allows for the suppression of one pathway while another is enhanced. A crucial component of this intricate system is the antigen-presenting cells (APCs), particularly dendritic cells (DCs), which link innate and adaptive immunity in the fight against viruses. These cells are also activated during BCG immunotherapy, initiating an immune response against cancer cells [11].

Secondly, viruses progress through various stages of infection. Cytomegalovirus (CMV), for instance, has a lytic (productive) and latent stage. Even during the productive stage, CMV expresses different segments of its genome as the infection advances, optimizing its environment step by step. In the latent stage, CMV remains dormant primarily in CD34+ stem cells, the precursors to DCs. The virus is reactivated when these DCs mature, such as during BCG immunotherapy [13].

Previous studies described improved survival in BC patients under BCG who produced anti-CMV immunoglobulin G (IgG) following treatment [14]. While primary CMV infection in its early stages can weaken this connection, the reactivation of dormant CMV may recruit T and natural killer (NK) cells to the site [15]. The rising anti-CMV IgG might represent a marker of successful DC maturation, which in turn reactivated dormant CMV. Moreover, the successful production of IgG and the activation of NK cells suggest an intact connection between DCs and the adaptive immune system.

**Figure 1:**
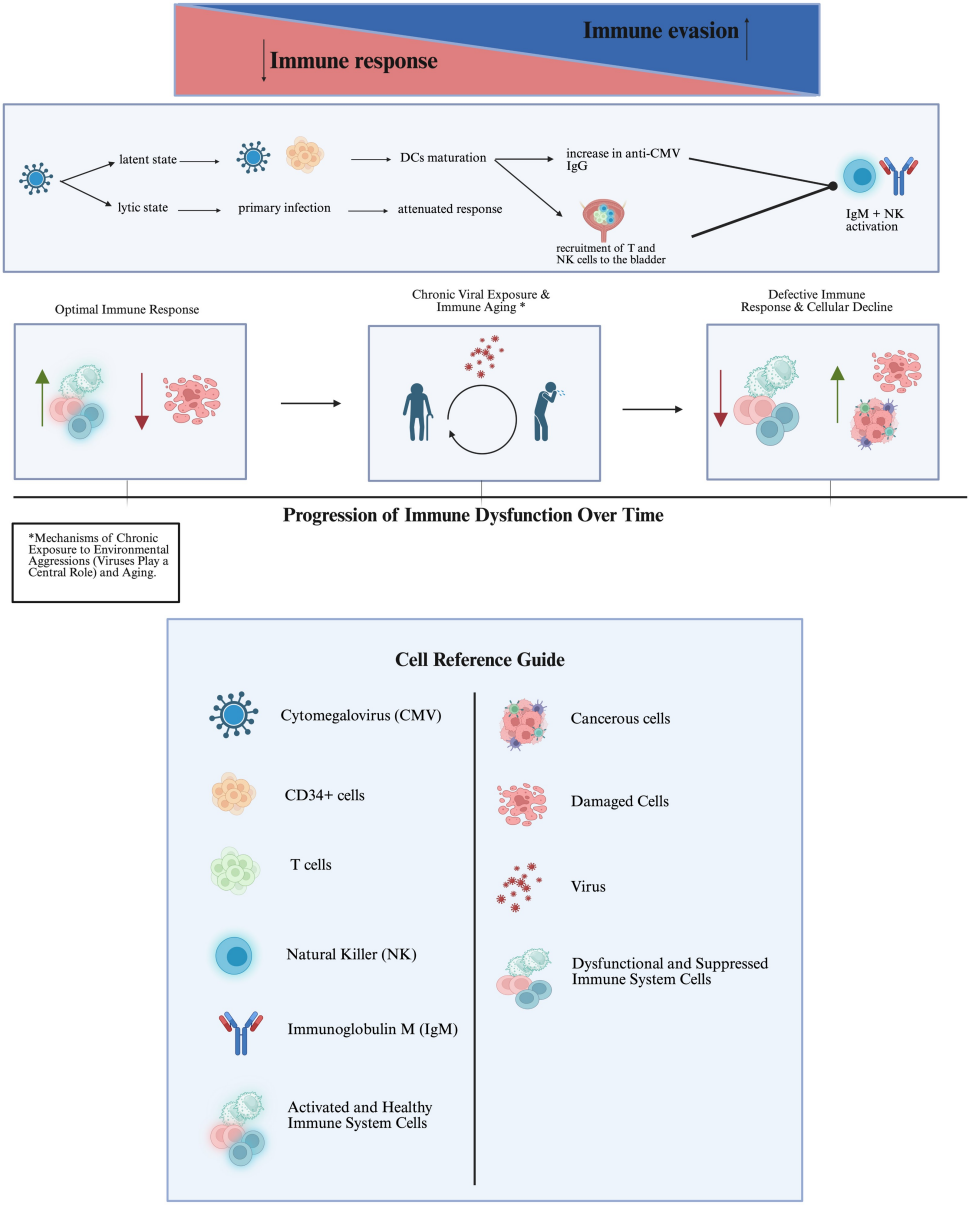
**Modulation/efficiency of the immune response: environmental factors, aging, and chronic exposure mechanisms**. The upper part: Created in BioRender. Bitencourt Pascoal, L. (2025) https://biorender.com/8vidydm; the lower part: Created in BioRender. Bitencourt Pascoal, L. (2025) https://biorender.com/qhkm6ia

Figure 1 shows potential mechanisms of immune response modulation, the role of environmental factors, aging, and chronic exposure mechanisms.

## Mechanisms of immune suppression by viruses

In 1986, Rouse and Horohov [16] described four mechanisms by which viruses impede immune responses: proliferation within all or specific subtypes of lymphocytes, production of immune suppressors, proliferation within phagocytic cells, and shifting the immune system into a regulatory state.

Recently, additional mechanisms such as immune exhaustion [17], immunosenescence [18], and clonal expansion of overly specific memory cells have been identified [19]. During primary infection, CMV’s genome can produce factors that inhibit DC maturation, thereby hindering the surface expression of T cell costimulatory molecules CD80 and CD86, reducing DC interleukin (IL) production, and preventing their migration to the lymph nodes. In the long term, continuous exposure to CMV antigens can lead to T cell exhaustion and senescence [19].

## Mechanisms of enhanced inflammation by viruses

During early infection, if CMV fails to disrupt the connection between DCs and the adaptive immune system, specific T cells may rapidly expand, leading to an infectious mononucleosis-like disease [20]. These T cells are not exclusively specific to CMV and have been shown to provide heterologous immunity against non-CMV viruses [21]. Additionally, CMV is associated with chronic inflammation by activating the NF-κB pathway and triggering pattern recognition receptors (PRRs) in the innate immune system [1].

Viruses can also induce inflammation by killing their host cells. This type of cell death, known as immunogenic cell death (ICD), has recently become a target for cancer immunotherapy. Coxsackievirus, adenovirus, Newcastle disease virus, and vesicular stomatitis virus are among the agents being investigated for their ICD potential against BC [22, 23]. In general, ICD is a type of regulated cell death that promotes the activation of innate and adaptive immune responses and immunological memory in infectious and malignant diseases.

Oncolytic immunotherapy has emerged as a promising strategy in cancer treatment, leveraging viruses to selectively infect and kill tumor cells while stimulating an immune response [23, 24]. Oncolytic immunotherapy, which relies on viruses’ selective infection and lysis of cancer cells, can induce ICD by stimulating the immune system to recognize and target tumor cells. Recent studies have shown that oncolytic viruses (OVs), upon infecting tumor cells, can trigger the release of damage-associated molecular patterns (DAMPs) and activate PRRs on immune cells, such as DCs. This activation of DCs leads to their maturation and subsequent priming of specific T cells that are crucial for antitumor immunity [24].

The ability of OVs, like coxsackievirus and adenovirus, to induce ICD has been explored as a promising approach for BC treatment. Moreover, these viruses induce a direct cytotoxic effect on tumor cells and stimulate a broader immune response, enhancing the overall inflammatory milieu in the TME. This provides a dual mechanism of action that may lead to improved therapeutic outcomes when combined with other immunotherapies [24, 25].

More specifically, recent studies have demonstrated that the immunogenicity of cancer cells is directly related to the production of tumor neoantigens, which are not recognized by central or peripheral tolerance mechanisms, and fetal antigens, which are generally absent in adult tissues. These antigens are processed and presented on the cell surface through major histocompatibility complex (MHC) class I and II molecules, enabling recognition by the immune system [26].

However, for this presentation to result in an effective immune response, the presence of costimulatory signals provided by APCs, such as DCs, is essential [26, 27]. ICD plays a crucial role in this process, as it triggers the release of “eat me” and “find me” signals, collectively known as DAMPs [27, 28]. These signals facilitate the recruitment and activation of DCs through their PRRs, leading to their maturation and subsequent cross-priming of CD8+ T cells. Among the key ICD-related DAMPs, the exposure of calreticulin (CALR) on the plasma membrane serves as an essential “eat me” signal by interacting with the LDL receptor-related protein 1 (*LRP1*) on DCs [26, 28].

Other signals, such as the autophagy-dependent secretion of adenosine triphosphate (ATP) and the passive release of annexin A1 (ANXA1), further enhance APC recruitment and facilitate immune activation [27, 28]. Additionally, the nuclear release of high-mobility group box 1 (HMGB1) binds to Toll-like receptor 4 (TLR4) on DCs, enhancing antigen cross-presentation and promoting CD8+ T cell activation [29].

Beyond these molecular events, the activation of innate immune pathways further amplifies the inflammatory response within the TME. The accumulation of mitochondrial DNA in the cytosol activates the cyclic GMP-AMP synthase (cGAS)-STING pathway, releasing type I interferons (IFN-Is) and other pro-inflammatory cytokines that strengthen adaptive immunity [28]. The exposure of heat shock proteins (HSPs), such as HSP70 and HSP90, on the surface of dying cancer cells also contributes to ICD, as these chaperones facilitate antigen presentation and immune activation [27, 28].

Furthermore, the phosphorylation of the eukaryotic translation initiation factor 2 subunit α (eIF2α) has been correlated with CALR exposure and tumor infiltration by DCs and CD8+ T cells, highlighting a potential evolutionary link between antiviral immune responses and cancer immunosurveillance [29, 30]. However, the effectiveness of ICD in eliciting robust antitumor immunity is often counteracted by immunosuppressive factors within the TME, such as infiltration by regulatory T cells (Tregs) expressing CTLA-4 and secreting IL-10 [30, 31].

Moreover, the fibrotic response observed in certain tumors, such as pancreatic ductal adenocarcinomas (PDACs), can hinder the mobility of primed T cells, limiting their ability to reach and eliminate tumor cells [30, 31]. Given these complexities, strategies aimed at modulating the TME and overcoming immune evasion mechanisms are essential to enhance the therapeutic efficacy of ICD-inducing agents in cancer treatment [27, 30, 31].

Thus, ongoing preclinical and clinical studies are focused on refining the use of OVs, addressing challenges like efficient delivery, immune evasion, and precise tumor targeting [32, 33]. The goal is to optimize therapeutic outcomes and overcome barriers to treatment success. This dual mechanism of oncolysis combined with immune activation holds significant promise for advancing cancer therapies and improving patient prognosis [25, 32].

## Viral infection modulates the immune response to cancer

Viruses have long been recognized for their ability to cause cancer, with certain oncoviruses directly contributing to tumor formation. Some studies estimate that viruses are responsible for approximately 10% to 15% of all cancers globally [25, 33].

For instance, human papillomavirus (HPV) infects epithelial cells and integrates its DNA into the host genome, leading to the expression of viral oncoproteins E6 and E7. Over time, this can lead to the development of malignant tumors, most commonly in the cervix, but also in the oropharynx, anus, and other genital areas [34].

However, beyond their direct oncogenic potential, viral infections whether past, latent, or associated with chronic disease can influence immune responses, contributing to immune evasion and the progression of more aggressive cancer forms. Recent studies have highlighted the critical role of the virome in shaping the TME and affecting the efficacy of cancer immunotherapies [35].

Viruses can modulate immune responses, influencing both the progression of cancer and the effectiveness of the immune system’s ability to detect and eliminate tumor cells. Latent viral infections, in which viruses persist in a dormant state within host cells, pose a unique challenge. These viruses can evade immune surveillance, alter cellular functions, and contribute to chronic inflammation factors that can all play a role in cancer development and immune response [36].

A key aspect of this interaction is the complex relationship between the virome and tumor-infiltrating T cells, which are central to the body’s anti-cancer immune response. Even at subclinical levels, persistent viral antigens can push T cells into dysfunctional states, undermining the immune system’s capacity to combat tumors effectively [37]. Two major dysfunctional states of T cells, exhaustion and senescence, are particularly relevant in this context.

T cell exhaustion, frequently observed in cancer, is characterized by the increased expression of multiple inhibitory receptors, such as programmed cell death 1 (PD-1) and T cell immunoglobulin and mucin domain 3 (Tim-3). These inhibitory molecules dampen effective antitumor immune responses, aiding tumor progression. Exhausted T cells exhibit a significant reduction in producing key cytokines like IL-2, IFN-γ, and TNF-α, alongside cell cycle arrest, collectively defining the exhausted phenotype [38]. In BC, T cell exhaustion has been implicated as a potential mechanism behind tumor recurrence following BCG therapy [39].

Persistent viral infections are generally associated with a weakened T cell response, primarily due to the exhaustion or depletion of virus-specific CD8+ T cells. This exhaustion process in chronic viral infections mirrors the dysfunction observed in tumor-specific CD8+ lymphocytes [40].

Senescent T cells, in contrast, are marked by reduced expression of costimulatory molecules CD27 and CD28 and the presence of senescence-associated markers CD57 and killer cell lectin-like receptor subfamily G member 1 (KLRG1), which are linked to telomere shortening. Unlike exhausted T cells, senescent T cells adopt a pro-inflammatory profile, secreting high levels of cytokines such as IFN-γ and IL-6 upon stimulation [41].

Senescence, often triggered by chronic or recurrent viral infections, results in a loss of proliferative capacity in T cells. While senescent T cells can generate pro-inflammatory signals, they are largely ineffective in mounting a robust antitumor response [18].

Dysfunctional T cells within the TME promote tumor development and undermine effective antitumor immunity, limiting the success of immunotherapies. Environmental factors, such as the antigenic burden from persistent viral infections and damage induced by reactive oxygen species (ROS), contribute to the premature onset of senescence [18].

In the case of BCG therapy for BC, the treatment induces intracellular oxidative stress, leading to direct cytotoxic effects. BC cells internalize BCG, which subsequently stimulates inducible nitric oxide synthase (iNOS) to produce nitric oxide (NO). Additionally, BCG therapy elevates ROS production, further amplifying NO levels and contributing to a cytotoxic microenvironment. While intended to target cancer cells, this oxidative stress may also exacerbate T cell exhaustion and senescence, further complicating the immune response and potentially contributing to BCG refractory bladder tumors [5]. Table 1 describes viral infections and their impact on BC immunotherapy.

**Table 1:** Viral infections and their impact on bladder cancer (BC) immunotherapy

**Virus**	**Effect on BC immunotherapy**	**Oncogenic mechanisms**	**References**
Cytomegalovirus (CMV)	May affect immune responses related to cancer, including tumor-promoting inflammation, immune evasion, and immunosuppression	CMV reactivation can recruit T and natural killer (NK) cells to the site of infection, promoting dendritic cell (DC) maturation and increasing the immune response against the tumor	[13, 14, 44, 45]
		Persistence can lead to T cell senescence, compromising immune effectiveness, especially in the tumor microenvironment (TME)	[44]
		Reactivation in bladder tissue after treatment, including infection of monocytes, fibroblasts, and tumor cells, indicating a complex role in cancer resistance and treatment responses	[45]
Epstein-Barr virus (EBV)	Potential to enhance immune responses against tumor-associated antigens (TAAs), but also associated with oncogenesis	EBV reactivation can stimulate T cell responses specific to TAAs, but the relationship between EBV and bladder carcinogenesis is not fully understood	[51, 52]
		In non-invasive muscle urothelial tumors, it was found that poor differentiation was correlated with a high genomic load of the EBV	[52]
		Latency within B cells with potential reactivation under immunosuppression	[50]
		Latent membrane protein 1 (LMP1) protein of EBV can regulate TAAs and stimulate strong CD4+ cytotoxic T lymphocyte (CTL) responses	[51]
		EBV-associated malignancies include nasopharyngeal carcinoma and Hodgkin’s lymphoma	[52]
Human papillomavirus (HPV)	Rarely involved in the pathogenesis of BC, but some studies suggest occasional involvement. Not directly discussed, but may impact immune modulation due to its oncogenic nature	HPV infection may contribute to tumor formation and affect the immune response to tumor cells, although its specific impact on Bacillus Calmette-Guerin (BCG) immunotherapy is unclear	[34, 36]
		HPV16 was found in a single case of BC, with no detection of HPV18. Previous research failed to detect HPV DNA in hundreds of bladder tumor samples	[46]
		A 2011 meta-analysis showed that 17% of BC cases were HPV-positive	[49]
BK polyomavirus (BKPyV)	It can interfere with the effectiveness of BCG treatment	BKPyV reactivation can affect local immunity and contribute to cancer progression, interfering with the effectiveness of BCG treatment, especially in immunocompromised patients	[53]
		One study detected BKPyV in 1.7% of BC biopsy specimens	[54]
	Possible involvement in early carcinogenesis and aggressive BC behavior	Cell proliferation in urothelial cells through large T antigen (LTag)	[55]
		Loss of p53 helicase domain in tumors, selecting for tumor survival	[56]
		Integration of viral and host genomes leads to “super-enhancers” that regulate gene expression, promoting cell cycle progression, DNA repair, and mitosis while downregulating cell adhesion genes	[59]
		Tumors exhibit invasive and high-grade behavior	[60]
John Cunningham polyomavirus (JCPyV)	Possible oncogenic potential, particularly in the development of BC	Persistent infection in kidneys and urinary tract after initial infection, with continuous oncogenic risk in urothelial cells	[55]
		Case reported in kidney transplant recipient with high-grade urothelial carcinoma after JCPyV nephropathy	[56]
		Potential for tumor cell transformation	[57]
Torque Teno virus (TTV)	No direct link to carcinogenesis, but may serve as an indicator of immune competence	Decreased presence in urine of BC patients compared to healthy volunteers	[58, 59]
		Highly prevalent but non-pathogenic	

## Antigenic burden and persistent infections

The lifelong burden of antigenic exposure, including persistent viral infections, has been suggested as a key factor in driving immunosenescence. Persistent infections and aging are also linked to developing the T cell exhaustion phenotype, further complicating the immune landscape in cancer patients [42]. Some persistent viruses, such as CMV, and other less frequent viruses present potential connections to BC.

Human herpesviruses (HHVs) are latent in the host and can affect cancer-related immune responses, such as tumor-promoting inflammation, immune evasion, and immunosuppression. CMV is a persistent herpesvirus that remains latent in the body after the initial infection, often reactivating under conditions of immune suppression. Affecting 60–90% of the global population, CMV remains latent throughout the host’s life and can cause severe complications in immunocompromised individuals [43].

CMV persistence is associated with T cell memory inflation, a state characterized by the continuous expansion and maintenance of CMV-specific CD8+ T cells, which remain functional and capable of responding to CMV antigens. Despite this, evidence suggests CMV persistence may also drive T cell senescence. This senescence can compromise the immune system’s effectiveness, particularly within the TME [44]. A recent study has highlighted CMV reactivation in bladder tissues following treatment, including infection of monocytes, fibroblasts, and tumor cells, indicating a complex role for CMV in cancer resistance and treatment responses [45].

The potential role of oncogenic viruses in the development of urothelial BC (UBC) has been the subject of several studies, particularly regarding the involvement of HPV. An analysis of 689 samples from patients with UBC between 2005 and 2011 identified HPV16 in only one case and found no detection of HPV18, suggesting that these viruses are rarely involved in the pathogenesis of UBC [46].

These findings are supported by previous research that failed to detect HPV DNA in hundreds of tumors analyzed by PCR [47, 48]. However, a 2011 meta-analysis reported that 17% of UBC cases were positive for HPV [49].

The relationship between HPV and BC development remains controversial in the literature, highlighting the need for further studies to clarify the influence of viral antigenic burden on the functional state of immune cells. So far, it is believed that part of these discrepancies stems from geographical variations in HPV prevalence in UBC, with higher incidences observed in regions such as Asia and Africa [50].

Different viruses might play various roles, mainly those with a latent phase. Epstein-Barr virus (EBV) is a ubiquitous herpesvirus that infects more than 90% of the global population. EBV is known for its ability to establish lifelong latency within B cells after primary infection and can reactivate under conditions of immune suppression. Although reactivation of the latent virus is a concern, long-term sequelae include malignancies such as nasopharyngeal carcinoma and Hodgkin lymphoma [50].

Recent studies have found that EBV latent membrane protein 1 (LMP1), when expressed in cancers unrelated to EBV, can upregulate tumor-associated antigens (TAAs) and stimulate a strong TAA-specific CD4+ cytotoxic T lymphocyte (CTL) response. This suggests that, besides its oncogenic role, EBV may have potential therapeutic applications in cancer treatment by enhancing immune responses against TAAs [51].

The relationship between urothelial carcinoma and the EBV virus is unclear. EBV DNA has been observed in bladder urothelial carcinoma specimens. In non-muscle-invasive urothelial tumors, poor differentiation was found to be correlated with a high EBV genome load [52].

The BK polyomavirus (BKPyV) is prevalent globally, with an incidence of about 80% for genotype I. After the initial infection, BKPyV remains latent in the urothelium and can be reactivated during periods of immunosuppression, establishing persistent infection. This leads to BKPyV-associated nephropathy after kidney transplantation and hemorrhagic cystitis [53]. BK viruria can be seen in 60% of kidney transplant recipients, while BK viremia is seen in up to 13% of kidney transplant recipients [54].

John Cunningham polyomavirus (JCPyV) is related to BKPyV, sharing 72% nucleotide homology and 80% identity in the amino acid sequence of the structural protein viral protein 1 (VP1). Approximately 70% of adults develop specific anti-JCPyV antibodies [55]. The virus has been linked to the development of BC due to its ability to transform cells and oncogenic potential. A recent study detected BKPyV and JCPyV in 1.7% and 6.1% of BC biopsy specimens, respectively [56].

The persistence of JCPyV in the kidney and urinary tract after initial infection and the continued oncogenic risk conferred by the virus in urothelial cells have gained considerable attention in the etiology of BC. Renal transplant recipients also have an increased risk of urothelial carcinoma. Although the role of the JCPyV virus in human cancer is not yet proven, the case of a kidney transplant recipient who developed high-grade urothelial carcinoma 5 years after the diagnosis of JCPyV nephropathy and 9 years after kidney transplantation has been reported [57].

Torque Teno virus (TTV), first discovered in 1997, is a highly prevalent, non-pathogenic virus with a notable presence in the human virome. TTV has attracted attention as a potential indicator of immunocompetence in solid organ transplant recipients. The role of TTV in carcinogenesis is still unknown, but recent work has shown a significant reduction in the presence of TTV in the urine of patients with BC when compared to healthy volunteers [58].

Immune suppression in organ transplant recipients increases the prevalence of viral infections, particularly from BKPyV, which is rarely observed in BC cases in the general population. BKPyV may play a key role in early carcinogenesis via clonal integration, driving tumor growth through its viral oncoproteins. BKPyV infection promotes cellular proliferation in urothelial cell culture through its large T antigen (LTag) [59].

In immunosuppressed BC individuals, nearly 50% of tumors contained viral sequences, primarily BKPyV (21%), JCPyV (16%), carcinogenic HPV (7%), and TTVs (12%). Like other virus-driven cancers, most BKPyV-positive tumors showed clonal viral genome integration linked to microhomology-mediated end joining and tumor genome amplifications. BKPyV tumors exhibited host gene expression changes consistently with LTag function. Mutation signatures linked to the apolipoprotein B mRNA editing enzyme catalytic polypeptide-like 3 (*APOBEC3*) and single base substitution 5 (*SBS5*) were the most frequent [59].

This research highlights the crucial role of viral integration in BC, particularly among immunocompromised organ transplant recipients, and underscores the contribution of human polyomaviruses and papillomaviruses to tumor development. Interestingly, BKPyV integration sites exhibit amplification of both viral and host genomes, potentially leading to the formation of “super-enhancers” that influence gene expression. In BKPyV-positive tumors, genes involved in cell cycle progression, DNA damage repair, and mitosis are upregulated alongside downregulated cell adhesion genes, contributing to their high-grade and invasive nature [60].

## Conclusions

The current study focuses on actionable clinical insights into the potential mechanisms of viral-induced immune modulation. Its goal is to identify patients who might benefit from specific screening or individualized treatment approaches in the future. Given the state of the science on the topic, there might be more questions than answers, considering that understanding the virome’s impact on the immune response and its clinical implications for cancer is a relatively new concept under investigation.

## Abbreviations

APCs: antigen-presenting cells
BC: bladder cancer
BCG: Bacillus Calmette-Guerin
BKPyV: BK polyomavirus
CMV: cytomegalovirus
DAMPs: damage-associated molecular patterns
DCs: dendritic cells
EBV: Epstein-Barr virus
HPV: human papillomavirus
HSPs: heat shock proteins
ICD: immunogenic cell death
IFN-Is: type I interferons
IgG: immunoglobulin G
IL: interleukin
JCPyV: John Cunningham polyomavirus
NMIBC: non-muscle invasive bladder cancer
OVs: oncolytic viruses
PRRs: pattern recognition receptors
TAAs: tumor-associated antigens
TME: tumor microenvironment
TTV: Torque Teno virus
UBC: urothelial bladder cancer

## Declarations

### Copyright

© The Author(s) 2025.

## References

[1] Stratton MR, Campbell PJ, Futreal PA (2009). The cancer genome. Nature.

[2] Dyrskjøt L, Hansel DE, Efstathiou JA, Knowles MA, Galsky MD, Teoh J (2023). Bladder cancer. Nat Rev Dis Primers.

[3] Sung H, Ferlay J, Siegel RL, Laversanne M, Soerjomataram I, Jemal A (2021). Global Cancer Statistics 2020: GLOBOCAN Estimates of Incidence and Mortality Worldwide for 36 Cancers in 185 Countries. CA Cancer J Clin.

[4] Bladder Cancer Stages [Internet]. https://www.cancer.gov/types/bladder/stages#_26.

[5] Han J, Gu X, Li Y, Wu Q (2020). Mechanisms of BCG in the treatment of bladder cancer-current understanding and the prospect. Biomed Pharmacother.

[6] Ibrahim OM, Kalinski P (2024). Breaking Barriers: Modulation of Tumor Microenvironment to Enhance Bacillus Calmette–Guérin Immunotherapy of Bladder Cancer. Cells.

[7] Malmström PU, Sylvester RJ, Crawford DE, Friedrich M, Krege S, Rintala E (2009). An individual patient data meta-analysis of the long-term outcome of randomised studies comparing intravesical mitomycin C versus bacillus Calmette-Guérin for non–muscle-invasive bladder cancer. Eur Urol.

[8] Zitvogel L, Ayyoub M, Routy B, Kroemer G (2016). Microbiome and Anticancer Immunosurveillance. Cell.

[9] Broecker F, Moelling K (2021). The Roles of the Virome in Cancer. Microorganisms.

[10] Libbey JE, Fujinami RS, Brogden KA, Guthmiller JM (2002). Virus-induced Immunosuppression. Polymicrobial Diseases.

[11] Jalalizadeh M, Dal Col LSB, Yadollahvandmiandoab R, Reis LO, Rezaei N (2022). BCG Immunotherapy: Old Tool and New Concepts. Handbook of Cancer and Immunology.

[12] Kipps A, Stern L, Vaughan G (1966). The duration and the possible significance of the depression of tuberculin sensitivity following measles. S Afr Med Assoc NPC.

[13] Sinclair J (2008). Manipulation of dendritic cell functions by human cytomegalovirus. Expert Rev Mol Med.

[14] Horowitz A, Guethlein LA, Nemat-Gorgani N, Norman PJ, Cooley S, Miller JS (2015). Regulation of Adaptive NK Cells and CD8 T Cells by HLA-C Correlates with Allogeneic Hematopoietic Cell Transplantation and with Cytomegalovirus Reactivation. J Immunol.

[15] Zangger N, Oxenius A (2022). T cell immunity to cytomegalovirus infection. Curr Opin Immunol.

[16] Rouse BT, Horohov DW (1986). Immunosuppression in viral infections. Rev Infect Dis.

[17] McLane LM, Abdel-Hakeem MS, Wherry EJ (2019). CD8 T Cell Exhaustion During Chronic Viral Infection and Cancer. Annu Rev Immunol.

[18] Reyes A, Ortiz G, Duarte LF, Fernández C, Hernández-Armengol R, Palacios PA (2023). Contribution of viral and bacterial infections to senescence and immunosenescence. Front Cell Infect Microbiol.

[19] Müller L, Di Benedetto S (2024). Immunosenescence and Cytomegalovirus: Exploring Their Connection in the Context of Aging, Health, and Disease. Int J Mol Sci.

[20] Ishii T, Sasaki Y, Maeda T, Komatsu F, Suzuki T, Urita Y (2019). Clinical differentiation of infectious mononucleosis that is caused by Epstein-Barr virus or cytomegalovirus: A single-center case-control study in Japan. J Infect Chemother.

[21] Sharma S, Thomas PG (2014). The two faces of heterologous immunity: protection or immunopathology. J Leukoc Biol.

[22] Yadollahvandmiandoab R, Jalalizadeh M, Buosi K, Garcia-Perdomo HA, Reis LO (2022). Immunogenic Cell Death Role in Urothelial Cancer Therapy. Curr Oncol.

[23] Jenkins E, Whitehead T, Fellermeyer M, Davis SJ, Sharma S (2023). The current state and future of T-cell exhaustion research. Oxf Open Immunol.

[24] Delaunay T, Achard C, Grégoire M, Tangy F, Boisgerault N, Fonteneau JF, Engeland CE (2020). A Functional Assay to Determine the Capacity of Oncolytic Viruses to Induce Immunogenic Tumor Cell Death. Oncolytic Viruses.

[25] zur Hausen H (1991). Viruses in human cancers. Science.

[26] Kroemer G, Galassi C, Zitvogel L, Galluzzi L (2022). Immunogenic cell stress and death. Nat Immunol.

[27] Chiaravalli M, Spring A, Agostini A, Piro G, Carbone C, Tortora G (2022). Immunogenic Cell Death: An Emerging Target in Gastrointestinal Cancers. Cells.

[28] Sprooten J, Garg AD, Kepp O, Galluzzi L Chapter Three - Type I interferons and endoplasmic reticulum stress in health and disease. International Review of Cell and Molecular Biology.

[29] Apetoh L, Ghiringhelli F, Tesniere A, Obeid M, Ortiz C, Criollo A (2007). Toll-like receptor 4-dependent contribution of the immune system to anticancer chemotherapy and radiotherapy. Nat Med.

[30] Fucikova J, Spisek R, Kroemer G, Galluzzi L (2021). Calreticulin and cancer. Cell Res.

[31] Togashi Y, Shitara K, Nishikawa H (2019). Regulatory T cells in cancer immunosuppression — implications for anticancer therapy. Nat Rev Clin Oncol.

[32] Hennessy ML, Bommareddy PK, Boland G, Kaufman HL (2019). Oncolytic Immunotherapy. Surg Oncol Clin N Am.

[33] de Martel C, Georges D, Bray F, Ferlay J, Clifford GM (2020). Global burden of cancer attributable to infections in 2018: a worldwide incidence analysis. Lancet Glob Health.

[34] zur Hausen H (2002). Papillomaviruses and cancer: from basic studies to clinical application. Nat Rev Cancer.

[35] Zhang J, Shi Z, Xu X, Yu Z, Mi J (2019). The influence of microenvironment on tumor immunotherapy. FEBS J.

[36] Liang G, Bushman FD (2021). The human virome: assembly, composition and host interactions. Nat Rev Microbiol.

[37] Moskophidis D, Lechner F, Pircher H, Zinkernagel RM (1993). Virus persistence in acutely infected immunocompetent mice by exhaustion of antiviral cytotoxic effector T cells. Nature.

[38] Wherry EJ (2011). T cell exhaustion. Nat Immunol.

[39] Strandgaard T, Lindskrog SV, Nordentoft I, Christensen E, Birkenkamp-Demtröder K, Andreasen TG (2022). Elevated T-cell Exhaustion and Urinary Tumor DNA Levels Are Associated with Bacillus Calmette-Guérin Failure in Patients with Non–muscle-invasive Bladder Cancer. Eur Urol.

[40] Wherry EJ, Blattman JN, Murali-Krishna K, van der Most R, Ahmed R (2003). Viral persistence alters CD8 T-cell immunodominance and tissue distribution and results in distinct stages of functional impairment. J Virol.

[41] Liu Z, Liang Q, Ren Y, Guo C, Ge X, Wang L (2023). Immunosenescence: molecular mechanisms and diseases. Signal Transduct Target Ther.

[42] Chou JP, Effros RB (2013). T cell replicative senescence in human aging. Curr Pharm Des.

[43] Griffiths P, Reeves M (2021). Pathogenesis of human cytomegalovirus in the immunocompromised host. Nat Rev Microbiol.

[44] Yu C, He S, Zhu W, Ru P, Ge X, Govindasamy K (2023). Human cytomegalovirus in cancer: the mechanism of HCMV-induced carcinogenesis and its therapeutic potential. Front Cell Infect Microbiol.

[45] Horowitz A (2024). Abstract IA007: Tumor HLA-E expression and cytomegalovirus infection modulate NK cell activity in human bladder cancer. Clin Cancer Res.

[46] Llewellyn MA, Gordon NS, Abbotts B, James ND, Zeegers MP, Cheng KK (2018). Defining the frequency of human papillomavirus and polyomavirus infection in urothelial bladder tumours. Sci Rep.

[47] Kao HL, Lai CR, Ho HL, Pan CC (2016). Molecular typing for detection of high-risk human papillomavirus is a useful tool for distinguishing primary bladder carcinoma from secondary involvement of uterine cervical carcinoma in the urinary bladder. Histopathology.

[48] Schmid SC, Thümer L, Schuster T, Horn T, Kurtz F, Slotta-Huspenina J (2015). Human papilloma virus is not detectable in samples of urothelial bladder cancer in a central European population: a prospective translational study. Infect Agent Cancer.

[49] Li N, Yang L, Zhang Y, Zhao P, Zheng T, Dai M (2011). Human papillomavirus infection and bladder cancer risk: a meta-analysis. J Infect Dis.

[50] Patel PD, Alghareeb R, Hussain A, Maheshwari MV, Khalid N (2022). The Association of Epstein-Barr Virus With Cancer. Cureus.

[51] Zhang Q, Xu M (2023). EBV-induced T-cell responses in EBV-specific and nonspecific cancers. Front Immunol.

[52] Chuang KL, Pang ST, Liao SK, Wu CT, Chang YH, Chuang HC (2011). Epstein-Barr virus DNA load in tumour tissues correlates with poor differentiation status in non-muscle invasive urothelial carcinomas. BJU Int.

[53] Chen Y, Trofe J, Gordon J, Du Pasquier RA, Roy-Chaudhury P, Kuroda MJ (2006). Interplay of cellular and humoral immune responses against BK virus in kidney transplant recipients with polyomavirus nephropathy. J Virol.

[54] Sharma R, Zachariah M (2020). BK Virus Nephropathy: Prevalence, Impact and Management Strategies. Int J Nephrol Renovasc Dis.

[55] Cayres-Vallinoto IM, Vallinoto AC, Pena GP, Azevedo VN, Machado LF, Ishak Mde O (2016). JC virus/human immunodeficiency virus 1 co-infection in the Brazilian Amazonian region. Braz J Infect Dis.

[56] Taherkhani R, Farshadpour F (2022). BK and JC polyomaviruses and risk of urothelial bladder carcinoma: a preliminary study in the northern shores of Persian Gulf, Iran. Infect Agent Cancer.

[57] Querido S, Fernandes I, Weigert A, Casimiro S, Albuquerque C, Ramos S (2020). High-grade urothelial carcinoma in a kidney transplant recipient after JC virus nephropathy: The first evidence of JC virus as a potential oncovirus in bladder cancer. Am J Transplant.

[58] Hrbáček J, Hanáček V, Kadlečková D, Cirbusová A, Čermák P, Tachezy R (2023). Urinary shedding of common DNA viruses and their possible association with bladder cancer: a qPCR-based study. Neoplasma.

[59] Starrett GJ, Yu K, Golubeva Y, Lenz P, Piaskowski ML, Petersen D (2023). Evidence for virus-mediated oncogenesis in bladder cancers arising in solid organ transplant recipients. Elife.

[60] Wang Y, Yan S, Liu Y, Yan Z, Deng W, Geng J (2023). Dynamic viral integration patterns actively participate in the progression of BK polyomavirus-associated diseases after renal transplantation. Am J Transplant.

